# Venom immunotherapy during COVID-19 pandemic: Experience from a University Allergy Center in Northern Italy

**DOI:** 10.1016/j.waojou.2020.100489

**Published:** 2020-11-13

**Authors:** Alessandro Dell’Edera, Franco Borghesan, Elisabetta Favero, Marcello Rattazzi, Riccardo Scarpa, Leonardo Tartaglia, Carlo Agostini, Francesco Cinetto

**Affiliations:** Allergy and Clinical Immunology Center, University of Padua, Department of Internal Medicine, Internal Medicine I Unit, Ca' Foncello Hospital, Treviso, Italy

**Keywords:** VIT, AIT, Hymenoptera, Covid-19, ARDS, VIT, Venom immunotherapy, ARDS, Acute respiratory distress syndrome, AIT, Allergen-specific immunotherapy, PD-1, Programmed cell death-1, CTLA-4, Cytotoxic T-lymphocyte antigen 4

## Abstract

During the ongoing pandemic of Coronavirus Disease 2019 (COVID-19) allergic patients need to continue their constant and proper treatment, including allergen-specific immunotherapy. These patients are expected to be at a higher risk for exacerbation of lung inflammation during viral infection. We investigated the putative interplay existing between allergen-specific immunotherapy and COVID-19 infection in a Hymenoptera venom–allergic population. We evaluated the frequency and severity of COVID-19 infection in a cohort of 211 subjects referring to our center for the regular administration of venom immunotherapy (VIT). Our result showed that the median age of our cohort is similar to the one that in our region has been associated with a high incidence of COVID-19 infection, increased hospitalization, and mortality rates. We reported only an isolated positivity of COVID-19 in the overall group; whereas none suffered from upper airway symptoms associated with COVID-19 (fever, cough, dyspnoea, sore throat, anosmia, and/or ageusia). Even though the demographic characteristics pose a substantial risk for such a population, we suggest that a regular administration of VIT may help in the development of an immunological milieu able to down modulate the Th1/Th17 environment that has been linked to inflammatory manifestations of COVID-19. To the best of our knowledge, this is the first description of the incidence of COVID-19 infection in Hymenoptera venom allergic patients treated with VIT, suggesting indirectly that venom immune tolerance-inducing treatment may be capable of reducing the aberrant inflammatory response induced by the virus in this specific population.

To the Editor

The ongoing pandemic of Coronavirus Disease 2019 (COVID-19) has become a global emergency for public health and medical communities.^1^ The severity of the disease ranges from an asymptomatic infection to an acute respiratory distress syndrome (ARDS) associated with Th1 and Th17 hypercytokinemia.^2^^,^^3^

Allergic patients and subjects suffering from chronic airway diseases were expected to be at a higher risk for exacerbation of lung inflammation during viral infection. Nonetheless, recent studies have shown a lower prevalence of allergic asthma in COVID-19 populations, suggesting that allergic diseases do not represent a potential comorbidity in the case of severe COVID-19-related diseases.^3^^,^^4^ This evidence has been observed even in allergic patients who needed a constant and proper treatment, including intranasal or inhaled corticosteroids, bronchodilators, biological therapuetics, and allergen-specific immunotherapy (AIT),^5^ a therapeutic modality which in our center has been continued in the majority of patients during the pandemic.

In this regard, limited data are available about the effect of AIT on COVID-19 infections, although it is well established that allergen-specific desensitization plays a role in the changes of cellular population and inflammatory signalling taking place in affected organs.^6^ As an example, there are experimental data suggesting that Hymenoptera venom immunotherapy (VIT) is able to sustain a suppressive milieu throughout the production of IL-10 and the induction of circulating regulatory T cells (Treg).^7^ Despite this, whether venom immune tolerance-inducing treatment is able to modulate the excessive inflammatory milieu that characterizes severe COVID-19-related ARDS is still unknown.

We evaluated the frequency and severity of COVID-19 infection in a cohort of Hymenoptera venom–allergic patients treated with VIT at our Allergy and Clinical Immunology Unit from the end of February until May 20, 2020. As shown in Table 1, a total sample of 211 patients was examined. The group consists of 171 males (81% of the total) and 40 females (19%), ranging in age from 11 to 85 (average age of 64.85 ± 10.05 years).

**Table 1:** Characteristics of the 211 patients referred to our Allergy Center for VIT.

Total subjects	n = 211
Vespa crabro	16
*Polistes*	63
*Vespula*	72
*Apis mellifera*	44
*Polistes* and *Vespula*	15
*Apis* and *Vespa crabro*	1
**Average age**	64.85 ± 10.05 y
**Contact with a positive subject**	53 (25%)
**ACE-i therapy**	24
**Median serum tryptase level**	8.13 ± 11.49 μg/L
**Health care professionals**	5

Total subjects of 211 patients referred to our Allergy center for VIT in the aforementioned frame of time, 16 of which are allergic to *Vespa crabro*, 63 to *Polistes*, 72 to *Vespula*, 44 to *Apis mellifera*, 15 to both *Polistes* and *Vespula* and one patient to both *Apis* and *Vespa crabro*. The average age was 64.85 ± 10.05 years. 24 patients are in mono-therapy with angiotensin-converting enzyme inhibitors (ACE-i). 5 subjects work as health care professionals

Following strict evaluation of eventual positivity of COVID-19 swab (according to our preventive measures for COVID-19 contagion), all subjects accessed the Allergy Unit of the hospital every 4–10 weeks for the VIT administration. Each patient completed a form with information regarding symptoms (fever, cough, dyspnoea, sore throat, anosmia, and/or ageusia) and eventual close COVID-19 contacts in the previous 14 days. None demonstrated to be suffering from upper airway symptoms associated with COVID-19. Fifty-three out of 211 patients (25%) confirmed the eventuality of contact with a positive subject. An isolated case of COVID-19 positivity was registered in a patient with preexisting cardiovascular disease and working as a health care professional who developed bilateral COVID-19-related pneumonia. The subject required hospitalization, an antiviral treatment (lopinavir/ritonavir), and hydroxychloroquine together with high-flow nasal cannula oxygen therapy and anti-IL-6 therapy (tocilizumab 8 mg/kg, two injections 12 hours apart). Upon treatment, the patient had a complete recovery in clinical laboratory parameters and lung imaging.

A considerable share of 171 subjects (81%) showed no evidence of a potential professional exposure, while 5 patients resulted to be health workers, thus being virtually at a higher biological risk; as mentioned above, 1 of these subjects developed COVID-19 associated pneumonia. About a half of our patients were retirees (48%) showing a median age of 69 years (between 65 and 75 years) which in our region has been associated with a high incidence of COVID-19 infection and increased hospitalization and mortality rates. Specifically, the average age of subjects who tested positive at COVID-19 swab and required hospitalization in the geographic area of Treviso was 72 ± 14.5 years, comprising 37 health care professionals (3.9%). Concerns exist that angiotensin-converting enzyme inhibitors (ACE-i) and angiotensin receptor blockers increase susceptibility to SARS-CoV-2 and the likelihood of severe COVID-19 illness. As shown in Table 1, in our cohort 24 patients were in monotherapy with ACE-i, but none of these patients developed COVID-19 disease. In our cohort the median serum tryptase level at baseline was 8.13 ± 11.49: no correlations were found between tryptase levels and COVID-19 infection.

Among its main effects, VIT contributes to an overall immunological downregulation secondary to the expression of IL-10 and the increase in circulating Treg cells, i.e. Foxp3+ CD4^+^ T cells that have been associated with a venom-specific IgG4/IgE antibody ratio shift capacity and with a modulatory role. In particular, a subpopulation of B cells — the so-called Breg cells — regulates excessive inflammation via the release of IL-10, inhibits proinflammatory response and increases IgG4 production.^8^ Indeed, programmed cell death-1 (PD-1) and cytotoxic T-lymphocyte antigen 4 (CTLA-4) expression on Treg cells are known to be involved in allergen tolerance.^9^ On the other hand, COVID-19 infection has been linked to the Th1/Th17 response and the inflammasome activation leading to a cytokine storm, ie, the immunological mechanism underlying ARDS.

Only one patient in the overall group had a mild course of COVID-19-related inflammatory syndrome, even though the demographic characteristics pose a substantial risk for such a population living in a geographical area where hospitalization and mortality associated to COVID-19 were higher in comparison to the national sample. Our data represent the first description of the incidence of COVID-19 infection in Hymenoptera venom allergic patients treated with AIT and our results indirectly propose that VIT may represent a putative protective factor in this specific population. We suggest that a regular administration of VIT may have an immunoregulatory role, favoring the development of an immunological milieu able to down modulate the Th1/Th17 environment. The greater limitation of a population study conducted on VIT patients lies mainly in the inclusion criteria for allergen-immunotherapy itself. As a matter of fact, VIT is generally proposed to healthy individuals since its contraindications among others list neoplastic diseases and autoimmune disorders non-responding to treatment. However, the study has the merit of sampling a cohort with a median age that constitutes a major risk factor for inflammatory manifestations of COVID-19,^4^ pointing out that VIT may be capable of reducing the aberrant immunological response induced by the virus even in an elderly population.

On this basis, we are planning to conduct an extensive retrospective data analysis of allergic patients referring to our center and undergoing any kind of allergen-specific immunotherapy in order to investigate if an interplay exists between desensitizing immunotherapy and COVID-19 resistance.

## Funding

No funding to declare.

## Authors' contributions

A.D: provided the design of the study, supplied the acquisition of data, drafting of manuscript and analysis and interpretation; C.A. and F.B.: provided the conception and design of the study, acquisition of data, analysis and interpretation of data, revised it critically for important intellectual content and final approval of the version to be submitted; F.C., R.S., M.R., L.T and E.F.: supplied the design of study and gave final approval of the version to be submitted.

## Ethics approval

Approval was obtained from the local ethics committee.

The data that support the findings of this study are available on request from the corresponding author, A.D. The data are not publicly available due to their containing information that could compromise the privacy of research participants.

## Consent for publication

Authors provided formal written Consent to Publish.

## Competing interests

The authors declare that there is no conflict of interest.
